# Aged garlic attenuates neuroinflammation via modulating the NF-κB pathway: Insights from multi-omics analyses

**DOI:** 10.29219/fnr.v69.11923

**Published:** 2025-07-09

**Authors:** Junjun Meng, Chengquan Wen, Xiaofan Fan, Jinxiu Guo, Shiyuan Zhao, Wenxue Sun, Wenxiu Han, Pei Jiang

**Affiliations:** 1 Translational Pharmaceutical Laboratory, Jining NO.1 People’s Hospital, Shandong First Medical University, Jining, China; 2 Institute of Translational Pharmacy, Jining Medical Research Academy, Jining, China; 3 Department of Pharmacy, Qingdao Eighth People’s Hospital, Qingdao, China

**Keywords:** aged garlic, neuroinflammation, omics analysis, network pharmacology, NF-κB pathway

## Abstract

**Background:**

Neuroinflammation is a key pathological feature in many neurodegenerative diseases, and the nuclear factor kappa-B (NF-κB) signaling pathway is a central mediator of this response. Aged garlic extract (AGE) is a functional food with well-documented antioxidant and anti-inflammatory properties, but its role in mitigating neuroinflammation remains unclear.

**Objective:**

This study investigates the effects of AGE on neuroinflammation by modulating the NF-κB signaling pathway using multi-omics analyses and experimental validation.

**Design:**

Lipopolysaccharide (LPS)-induced BV2 microglial cells and LPS-treated C57BL/6 mice were used to assess the effects of AGE. Transcriptomics, metabolomics, and network pharmacology approaches identified potential targets and pathways, focusing on NF-κB signaling. In vitro and in vivo models were employed to evaluate behavioral, biochemical, and histological outcomes.

**Results:**

AGE reduced pro-inflammatory cytokines (tumor necrosis factor-α, interleukin-1β, inducible nitric oxide synthase, and cyclooxygenase-2) in LPS-stimulated BV2 cells and suppressed microglial activation and neuronal damage in LPS-induced mice. Transcriptomic analysis showed that NF-κB pathway inhibition mediated these effects, with molecular docking confirming interactions between aged garlic compounds and NF-κB targets (NF-κB2 and NF-κB3).

**Conclusion:**

AGE attenuates neuroinflammation by inhibiting the NF-κB signaling pathway, improving cognitive and motor functions, and reducing neuronal injury in experimental models. These findings suggest aged garlic as a promising neuroprotective agent against neuroinflammation.

## Popular scientific summary

- Combines transcriptomics, metabolomics, and network pharmacology to identify aged garlic’s anti-neuroinflammatory mechanisms.
- Confirmed anti-inflammatory effects in both LPS-stimulated BV2 microglial cells and a mouse neuroinflammation model.
- Both ethyl acetate (lipophilic) and water (polar) extracts of aged garlic demonstrated antiinflammatory effects.

Garlic (*Allium sativum L*.) is known as a traditional ‘medicine food homology’ plant that is used both for food and medicine. It is the earliest recorded medicinal plant, and its great value has been recognized since ancient times (1, 2). Researchers have demonstrated that garlic contains various nutrients and has neuroprotective effects against neurofunctional damage caused by multiple factors (3–5). The aforementioned reports have demonstrated that garlic, being a representative agricultural commodity, possesses significant potential in the therapeutic management of neurological disorders.

Our previous research has shown that different varieties of garlic exhibit certain differences in their metabolites (6). Despite the various advantageous properties of garlic for human health, its distinctively strong odor and taste can lead to gastrointestinal discomfort, thereby hindering its widespread acceptance. However, this limitation has been addressed through the development of aged garlic (AG). AG is produced by subjecting fresh garlic to specific temperature and humidity conditions for a designated duration, resulting in a blackened appearance. Numerous studies indicate that aged garlic extract has extensive outcomes on antibacterial, antidiabetic, antioxidant, hypoallergenic, anti-inflammatory, hypocholesterolemic, and anti-carcinogenic activities (7). The primary impact of AGE is its role as an antioxidant, as it effectively enhances the antioxidant effects and other cellular antioxidants within vascular endothelial cells through the elimination of reactive oxygen species (8). According to the reports, AGE has demonstrated the ability to mitigate injury caused by reactive oxygen species and protect against brain injury, resulting in a reduction in infarct size and exhibiting a neuroprotective impact on cerebral ischemia (9–11). Despite the superior pharmacological properties of AGE compared to garlic, there remains a scarcity of literature regarding the active constituents and neurological effects of AG, and the precise anti-neuroinflammatory effects of AG remain ambiguous.

As a fermented derivative of garlic, AG effectively eliminates the pungent odor associated with fresh garlic and instead imparts a pleasant, smooth, and waxy texture. AG, a product of fermented fresh garlic, may undergo compositional changes due to chemical reactions during fermentation. Therefore, in this study, we first used metabolomics methods to analyze the differential metabolites between AG and fresh garlic, selecting representative components and those significantly upregulated in AG for network pharmacology analysis. Network pharmacology was utilized in this study to forecast the synergistic effects of AG against neuroinflammation, targeting multiple pathways and targets. Initially, the effective constituents of AG were identified based on specific screening criteria, and the core targets associated with both AG and neuroinflammation were selected using the protein–protein interaction (PPI) network. Subsequently, functional enrichment analysis using Gene Ontology (GO) and Kyoto Encyclopedia of Genes and Genomes (KEGG) was employed to identify the key signaling pathways involved in AG’s anti-neuroinflammatory effects. The effects of AG on neuroinflammation were preliminarily validated both in vivo and in vitro. A flowchart outlining the methodology employed in this research is provided.

## Methods and materials

### Sample preparation

The garlic samples were purchased from the local supplier. Based on the reports, both the aqueous extract and the polar solvent extract of AG contain a rich diversity of metabolites. Therefore, in this experiment, we used a 1:1 mixture of the water extract and the polar solvent ethyl acetate extract with the aim of achieving a more comprehensive analysis of all metabolites in AG. The garlic and AG were evenly divided into two portions. One portion was extracted three times using ethyl acetate with ultrasound, while the other portion was extracted three times with an equal amount of water using ultrasound. The extracts were then combined. After drying on a rotary evaporator, a 100 mg/mL solution with 50% (v/v) methanol was prepared. The obtained extracts were filtered through a 0.20 µm filter and stored at –20ºC until analysis.

### Metabolic analysis of AG and fresh garlic

LC-MS/MS analyses were performed using an UHPLC system (Vanquish, Thermo Fisher Scientific) with a UPLC HSS T3 column (2.1 mm × 100 mm, 1.8 μm) coupled to an Orbitrap Exploris 120 mass spectrometer (Orbitrap MS, Thermo). The mobile phase consisted of 5 mmol/L ammonium acetate and 5 mmol/L acetic acid in water (A) and acetonitrile (B). The autosampler temperature was 4°C, and the injection volume was 2 μL. The Orbitrap Exploris 120 mass spectrometer was used for its ability to acquire MS/MS spectra on the information-dependent acquisition mode in the control of the acquisition software (Xcalibur, Thermo). In this mode, the acquisition software continuously evaluates the full scan MS spectrum. The Electrospray ionization (ESI) ESI source conditions were set as following: sheath gas flow rate as 50 Arb, Aux gas flow rate as 15 Arb, capillary temperature 320°C, full Mass spectrometry (MS) resolution as 60,000, MS/MS resolution as 15,000 collision energy as 10/30/60 in NCE mode, spray voltage as 3.8 kV (positive) or –3.4 kV (negative), respectively.

To identify significant metabolites and visualize group separations, supervised orthogonal projections to latent structures-discriminant analysis (OPLS-DA) were utilized. A seven-fold cross-validation was then conducted to determine R2 and Q2 values, where R2 reflects the extent of variation explained by the model, and Q2 represents its predictive accuracy. To further assess the model’s robustness and predictive capacity, 200 permutations were performed, yielding intercept values for R2 and Q2. The Q2 intercept value serves as an indicator of model reliability, robustness, and the risk of overfitting, with lower values being preferable. Differentially expressed metabolites were identified with a significance threshold of *p* < 0.05 and |log2FC|>1. Pathway enrichment analysis was performed using commercial databases such as KEGG and MetaboAnalyst (http://www.metaboanalyst.ca/).

### Candidate compounds of AG

In the present study, AG was purchased from a local supermarket, and the chemical compounds data for AG were acquired from SymMap (http://symmap/org/) (12), which is one synthesis database that encompassed the information from the Traditional Chinese Medicine System Pharmacology (TCMSP) database and analysis platform (https://old.tcmsp-e.com/tcmsp.php) and the Encyclopedia of Traditional Chinese Medicine (ETCM, http://www.nrc.ac.cn:9090/ETCM/) database (13). The pharmacokinetic method Absorption, distribution, metabolism, excretion, and toxicity (ADMET) indexes were used to evaluate the drug similarity and pharmacokinetic characteristics of the candidate compounds. TCMSP was then used to further predict possible targets for these effective components. Then we used the Universal Protein Resource (UniProt, http://www.uniprot.org/) (14) to obtain the gene names of these targets.

### Identification of candidate targets of neuroinflammation

The standardization of target protein names for *Homo sapiens* was conducted using the Human Gene Database (GeneCards) and UniProt. The identification of disease-related targets involved the utilization of three databases, namely GenCards (https://www.genecards.org/), OMIM (https://www.omim.org/) database, CTD (https://ctdbase.org/) database and DisGeNET (https://www.disgenet.org/). The keyword ‘neuroinflammation’ was used for the search, and only ‘*Homo sapiens*’ proteins were selected. Finally, the overlap targets of compound and disease were obtained.

### Construction of the interaction network

These targets were collected and Cytoscape software (version 3.8.2) was used to obtain the compound–target–pathway interaction and PPI interaction network, set up on Metascape and STRING databases (https://string-db.org/).

### GO and KEGG pathway enrichment analysis

We used the DAVID (https://david.ncifcrf.gov/home.jsp) database to incorporate the identified potential targets for the treatment of neuroinflammation with AG into the roster of target gene names, with a focus on human species. The David database was employed to conduct an analysis of the enrichment of co-acting targets in terms of GO function (cellular, molecular, and biological) and the KEGG pathway.

### Molecular docking

The required files were obtained from the PDB database (PDB, https://www.rcsb.org/). All receptor files were opened in Pymol software to remove water molecules and add hydrogen and AutoDockVina was used for molecular docking to obtain validation results of molecular docking. The program performed a conformational search for the ligand in the box range and ultimately scored them based on their conformation, orientation, position, and energy using Vina. After Vina finished running, the binding affinity score between macromolecular receptors and small molecule ligands was output in the log.txt folder. The best combination mode was presented at the first line in log.txt with the lowest docking binding free energy. The interaction between the receptor and the ligand was visualized using discovery software.

### Cell culture and viability assay

The BV2 cells were purchased from the Cell Bank of the Chinese Academy of Sciences. Cell viability was detected by 3-(4,5-Dimethylthiazol-2-yl)-2,5-diphenyltetrazolium bromide (MTT) assay. A total of 5 × 10^3^ cells were inoculated in 96-well plates and pre-incubated with 0–50 μg/mL or 1% DMSO for 1 h and lipopolysaccharide (LPS) for 24 h. A volume of 20 μL MTT solution was added to each well (Shanghai Biyuntian Biotechnology Co., LTD., Shanghai, China). After incubation at 37°C for 4 h, 150 μL DMSO was added to each well, and the absorbance of formaldehyde was determined by BioTek at 570 nm.

### Animal experiments

Wild-type male C57BL/6J mice (6–8 weeks old, 20–25 g) were purchased from Jinan Pengyue Experimental Animal Breeding Co., LTD (Jinan, Shandong, China). Our animal protocols strictly followed the Guide for Care and Use of Laboratory Animals (Chinese Council) and ARRIVE guidelines, and they received ethical approval from the Jining NO.1 People’s Hospital (protocol number: JNRM2022DW071). The experimental diet composition, derived from the AIN93 M formula, is detailed in Table S1. Each mouse was housed individually in a pathogen-free environment with a 12-h light/dark cycle, in a temperature-regulated room maintained at 24 ± 2°C. Mice were divided into four groups (*n* = 25): Control group (daily administered 1 mL/kg of saline intraperitoneally), LPS group (daily administered 1 mg/kg LPS intraperitoneally), LPS + AGE (E) group (daily administered 50 mg/kg AGE ethyl acetate extract), and LPS + AGE (W) group (daily administered 50 mg/kg AGE water extract) according to the pre-experimental results 30 min after LPS intraperitoneal injection. Consequently, the two extracts were administered to mice via the intraperitoneal injection method for a duration of 7 days prior to conducting behavioral tests. Upon completion of the study period, all mice were euthanized and dissected under anesthesia following an overnight fast (12 h).

According to Hossein Zeinali (15), 50 mg/kg S-allyl cysteine from AGE administered orally (p.o.) improved the clinical and neuropathological features of experimental autoimmune encephalomyelitis in C57BL/6 mice. AGE (50 mg/kg) significantly reduced the latency time prolonged by scopolamine and increased the number of platform crossings. AGE (50 mg/kg) protected against scopolamine-induced cognitive impairment through decreasing oxidative damage and regulating cholinergic function in the brains of mice (16). Based on the results of this experiment, this study was conducted by administering an intraperitoneal injection at an intermediate concentration of 50 mg/kg for 14 days.

### Aged garlic extract

The extract was obtained as previously described. Briefly, the AG were evenly divided into two portions. One portion was extracted three times using ethyl acetate with ultrasound, while the other portion was extracted three times with an equal amount of water using ultrasound. After drying on a rotary evaporator, the obtained extracts were filtered through a 0.20 µm filter and stored at –20ºC until use. The chemical compositional analysis was measured using the High-Performance Liquid Chromatograph.

### Real-time quantitative PCR

Total Ribonucleic acid (RNA) was extracted from cells using the RNAex Pro reagent (Tsingke Biotech, China) according to the manufacturer’s protocol. RNA concentration was measured with a Nanodrop 2000C (Thermo Scientific). Genomic Deoxyribonucleic acid (DNA) (gDNA) was removed from the RNA samples, and the RNA was then reverse transcribed into cDNA using the Evo M-MLV RT Hybrid Kit (Tsingke Biotech, China). The cDNA was subsequently amplified using the SYBR® Green Premix Pro-Taq HS qPCR Kit (Tsingke Biotech, China) along with specific primers in the CFX96 real-time Polymerase Chain Reaction (qPCR) system (Bio-Rad, USA). Relative mRNA expression levels were calculated using the 2^−∆∆Ct^ method, with GAPDH used as the reference gene for normalization. The primers utilized in this study are detailed in Table S2.

### Western blot

After anaesthetizing with pentobarbital sodium, the chest cavity of the mouse is cut open to expose the heart. Next, tweezers should be utilized to elevate the apex of the heart and carefully insert the needle into the left ventricle. Subsequently, the right atrial appendage should be incised, and physiological saline should be gradually injected for perfusion until the lungs and liver exhibit a gray-white coloration. Following this, the mouse’s head should be detached, and the brain should be extracted. The hippocampal tissue of the mice was homogenized using the Radio Immunoprecipitation Assay (RIPA) buffer obtained from Thermos, Waltham, MA, USA. The resulting homogenate was then stored on ice for a duration of 30 min, followed by centrifugation at 4°C at a speed of 12,000×g for 15 min. Subsequently, the sample was supplemented with five times the loading buffer and subjected to boiling at 100°C for a period of 10 min. The total protein was separated by 10~15% SDS-PAGE. The membrane was incubated with 5% bovine serum albumin (BSA) at 25°C for 2 h and then incubated with a different primary antibody: anti-tumor necrosis factor-α (TNF-α) (#ab183218), anti-interleukin-1β (IL-1β) (#ab254360), anti-inducible nitric oxide synthase (iNOS) (#ab178945), anti-COX2 antibody (#ab179800), P65 (#AF5006), P-P65 (#AF2006), anti-IκB (#ab32518), P-IκB (#ab133462), GAPDH (#10494-1-AP) antibodies overnight at 4°C. Subsequently, the second antibody was incubated at room temperature for 1 h, and the film was subjected to analysis using a two-color infrared laser scanning imaging system to detect specific bands. Image J and GraphPad Prism 8 were used for quantitative analysis of the scanning results.

### Transcriptomics analysis

Using a Novaseq 6,000 platform, 150 bp paired-end reads were generated from the libraries. HTSeq-count4 was used to estimate FPKM3 and read counts for each gene. An analysis of principal component analysis (PCA) was conducted using R (v 3.2.0) to determine whether samples were biologically duplicated. *Q* value < 0.05 and foldchange > 2 or fold change < 0.5 was set as the threshold for significantly differential expression gene.

### Morris Water Maze test (MWM)

Before the experimental trials in the water maze begin, fill the pool with tap water and heat it, setting the platform 1 cm below the water surface. The water maze pool was partitioned into four quadrants, with one quadrant containing a submerged white platform with a diameter of approximately 2 cm, which remained fixed throughout the experiment. Non-toxic white temporary paint was used to make the water opaque. Facing the pool wall, the handler will place the animal in the water and then step back to a designated position to observe the animal completing the maze task. The mice were given a period of 4 days to get acclimatised to the test environment prior to commencing the hidden platform training. Each animal will undergo three consecutive trials. The animal was analyzed until it reaches the platform and the time taken was recorded. The procedure was initiated 7 days subsequent to LPS stimulation.

### Open field test in mice

The square open field box (50 × 50 × 35 cm) was divided into peripheral and central zones. The mice underwent a period of acclimation to the laboratory environment lasting a minimum of 2 days, with each day consisting of at least 4 h. Behavioral testing was conducted both prior to the formation of groups and 7 days following the administration of LPS. The SuperMazeTM software was utilized for the purpose of recording and analyzing the animals’ physical activity.

### Nissl staining

The specimens were embedded in paraffin, sliced and treated with a Nissl staining solution. Damaged neurons atrophy or contain vacuoles, while normal neurons have larger intact somatic cells and larger rounded nuclei.

### Immunofluorescence staining

Brains were removed and preserved in 4% Paraformaldehyde (PFA) solutions at 4°C for 24–48 h, and then soaked in 30% sucrose solutions for 72 h for them to dehydrate completely. Next, the brains were sliced into 8-μm sections for immunofluorescence staining. Immufluorescence was performed on the slides by reheating for 30 min at room temperature, rinsing three times with PBS, then blocking for 60 min at room temperature with QuickBlotTM Blocking Buffer. After incubation with primary antibodies (12 h, 4°C), anti-iba1 was applied. Subsequently, the slides underwent a second rinsing process and were then exposed to fluorescence-conjugated secondary antibodies that corresponded to the primary antibodies for a duration of 2 h at a temperature of 25°C (ab104139). The final step was to use a fluorescence microscope to take images (Olympus Co., Tokyo, Japan).

### RNA isolation and library preparation

Total RNA was extracted using the TRIzol reagent (Invitrogen, CA, USA) according to the manufacturer’s protocol. RNA purity and quantification were evaluated using the NanoDrop 2,000 spectrophotometer (Thermo Scientific, USA). RNA integrity was assessed using the Agilent 2,100 Bioanalyzer (Agilent Technologies, Santa Clara, CA, USA). Then the libraries were constructed using VAHTS Universal V6 RNA-seq Library Prep Kit according to the manufacturer’s instructions. The transcriptome sequencing and analysis were conducted by OE Biotech Co., Ltd. (Shanghai, China).

### RNA sequencing and differentially expressed genes analysis

Using a Novaseq 6,000 platform, 150 bp paired-end reads were generated from the libraries. HTSeq-count4 was used to estimate FPKM3 and read counts for each gene. An analysis of PCA was conducted using R (v 3.2.0) to determine whether samples were biologically duplicated. Differentially expressed genes (DEGs) were identified with a significance threshold of *p* < 0.05 and |log_2_FC|>1. Hierarchical cluster analysis of DEGs was performed using R (v 3.2.0) to demonstrate the expression pattern of genes in different groups and samples. The radar map of top 30 genes was drawn to show the expression of up-regulated or down-regulated DEGs using R packet ggradar .

### Statistical analysis

Data are expressed as mean ± standard deviation. Data analysis was performed using GraphPad Prism 8 (GraphPad, San Diego, CA, USA). One-way analysis of variance (ANOVA) was utilized, followed by Duncan’s multiple-range test to evaluate differences among the groups. The data were analyzed by one-way ANOVA. A *p*-value less than 0.05 was considered significant.

## Results

### Analysis of the differential metabolites

The literature reports that the components in garlic ethanol extract exhibit strong anti-biofilm activity, indicating that ethanol extraction can effectively release the active compounds (17) and prevents malignant evolution of non-invasive breast tumor cells induced by moderate hypoxia (18). The ethyl acetate fraction of AGE reduces cellular oxidative stress, enhances the viability of PC12 cells, and decreases cell death caused by Aβ-induced cytotoxicity (19). Other research also demonstrated that the distilled water extract of AGE contained significantly higher levels of total phenols and flavonoids compared to fresh garlic. Additionally, the 2,2-Diphenyl-1-picrylhydrazyl (DPPH), 2,2’-Azino-bis(3-ethylbenzothiazoline-6-sulfonic acid) (ABTS), Ferric reducing antioxidant power (FRAP), and H_2_O_2_ scavenging activities of the AGE extract were greater than those observed in fresh garlic (20). Therefore, in this study, to conduct a more comprehensive analysis of the components in AGE, we used two solvents with different polarities in equal volumes to extract the AG. The two extracts were then mixed in a 1:1 ratio for metabolics.

**Fig. 1:**
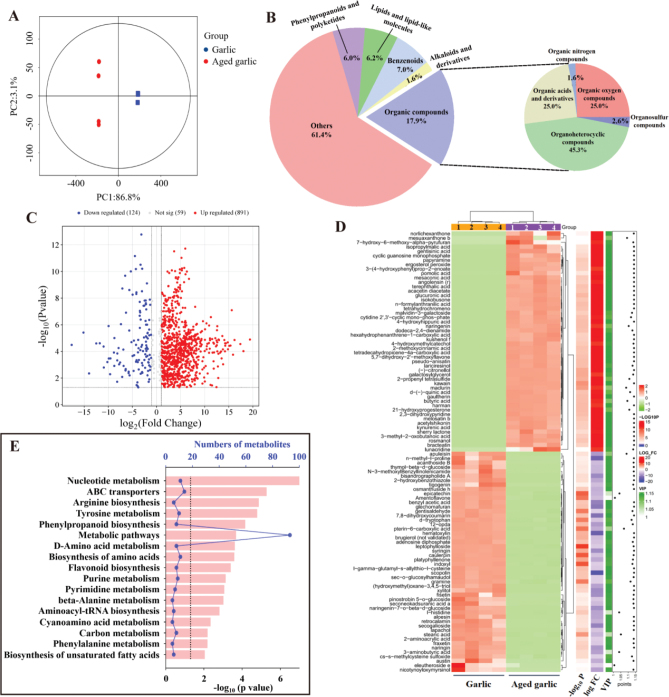
Differential metabolites between aged garlic and fresh garlic. (A) PCA plot among the two groups. (B) Classification pie chart of differential metabolites. (C) Volcano plots of differential metabolites in aged garlic and fresh garlic. (D) Heatmaps of the differential metabolites. (E) KEGG enrichment of 17 pathways related to inflammation. PCA: principal component analysis; KEGG: Kyoto Encyclopedia of Genes and Genomes.

PCA was employed to visualize the variability in the composition of extracts from AG and fresh garlic. The PCA plot revealed that the first and second principal components accounted for 86.8 and 3.1% of the total variance in the dataset, respectively (Fig. 1A). Untargeted metabolomics identified a total of 1,074 metabolites across these two garlic varieties. Differentially expressed metabolites were identified with a significance threshold of *p* < 0.05 and |log2FC|>1. These metabolites included lipids and lipid-like molecules (6.20%), phenylpropanoids and polyketides (6.0%), alkaloids and derivatives (1.60%), and benzenoids (7.0%). Organic compounds such as organosulfur compounds, organic nitrogen compounds, organic oxygen compounds, and organoheterocyclic compounds together made up 17.9% (Fig. 1B). Compared to fresh garlic, 891 metabolites were upregulated, while 124 were downregulated in AG (Fig. 1C). The differentially expressed metabolites primarily belonged to categories such as alkaloids, amino acids and peptides, carbohydrates, polyketides, shikimates and phenylpropanoids, terpenoids, and fatty acids (Fig. 1D). The metabolic pathways for these differential metabolites were obtained from the KEGG database, leading to the identification of 82 distinct metabolic pathways between AG and fresh garlic. Of these, 25 pathways showed significant differences (*p* < 0.05). The most notably enriched pathways included nucleotide metabolism, tyrosine metabolism, flavonoid biosynthesis, phenylpropanoid biosynthesis, and carbon metabolism (Fig. 1E).

Thus, during the fermentation process of fresh garlic into AG, the synthesis and metabolism of amino acids, the biosynthesis of flavonoids, phenylpropanoids, and unsaturated fatty acids occur. This process is also accompanied by the metabolism of phenylalanine, cyanoamino acids, nucleotides, and certain carbon compounds, ultimately resulting in the reduction of pungency and the development of a unique taste profile.

### Potential active components in AG and targets prediction

**Fig. 2:**
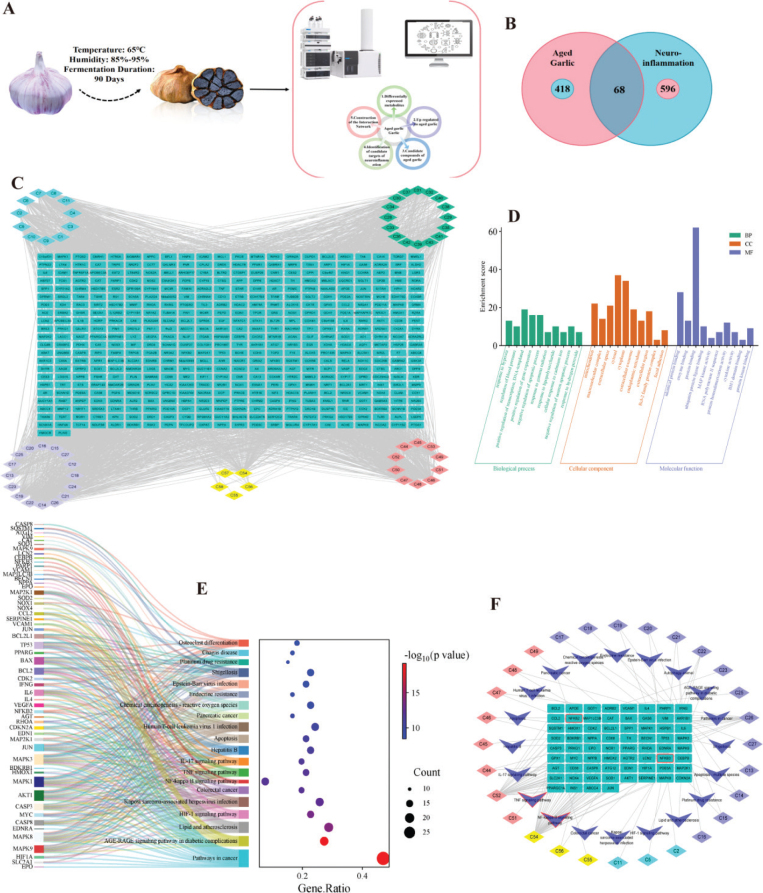
Network pharmacology uncovers the mechanism of aged garlic for neuroinflammation. (A) The procedure of the experimental. (B) Venn diagram of targets shared by aged garlic and neuroinflammation. (C) The components and potential targets network. (D) GO-enrichment analysis of common targets. (E) KEGG pathway analyses of the hub genes. (F) Aged garlic component–target–neuroflammation network. GO: Gene Ontology; KEGG: Kyoto Encyclopedia of Genes and Genomes.

Based on the metabolomic data, we selected the basic components of garlic which are common in AG and garlic, as well as the components whose expression was significantly up-regulated in AG compared with garlic, for network pharmacology studies (Fig. 2A). A total of 58 candidate compounds were identified by screening and excluding duplicates (Table S3). The 58 compounds are divided into four categories: 11 organic sulfides (such as alliin, diallyl disulfide, diallyl tetrasulfide, thiocyclohexane, etc.), 16 phenols (such as epigallocatechin, coumaric acid, rosmarinic acid, etc.), 16 amino acids and their derivatives (such as arginine, glutamine, aminobutyric acid, picric acid, etc.), and 10 kinds of flavonoids (flavonol, hesperidin, luteolin, etc.) and others (such as caffeine, berberine, etc.); a total of 486 target proteins were captured. The compound target interaction was established and visualized through Cytoscape 3.8.2 (Fig. 2C).

### Potential targets prediction of neuroinflammation and common targets

Using OMIM, MalaCards, and DisGeNET databases, the keyword ‘neuroinflammation’ was used to search for potential therapeutic targets related to neuroinflammation. After eliminating duplication, a total of 664 potential neuroinflammation treatment target genes were obtained. Using Venny 2.1 to screen common target genes for AG components and neuroinflammation, 68 common target genes were obtained (Fig. 2B). These included BCL2L1, CASP3, NFKB2, NFKB3, IL-4, interleukin-6 (IL-6), etc., which may be potential target genes for AG to exert neuroinflammation protective effects.

### GO and KEGG enrichment analysis

To investigate the potential mechanism of AG in combating neuroinflammation, an analysis of GO and KEGG enrichment was conducted on the aforementioned set of 68 core genes. Figure 2D lists the top 30 important GO terms and TOP 20 KEGG pathways (Fig. 2E). GO results showed that the BP terms with the highest levels of enrichment included response to lipopolysaccharide, response to hypoxia, positive regulation of gene expression, and so on. The CC category includes cytosol, macromolecular complex, and mitochondrion. In the MF category, the most enriched terms were enzyme binding and protein binding activity. The pathways depicted in Fig. 4E demonstrate potential avenues for investigation. Among these groups, the nuclear factor kappa-B (NF-κB) signaling pathway displayed the closest association with LPS-stimulated neuroinflammation. Based on the above drug target prediction, GO annotation, and KEGG pathway enrichment analysis, we speculate that AGE may regulate the expression of corresponding target genes through various bioactive substances, thereby regulating the NF-κB signaling pathway, and it is important in fighting neuroinflammation.

**Fig. 3:**
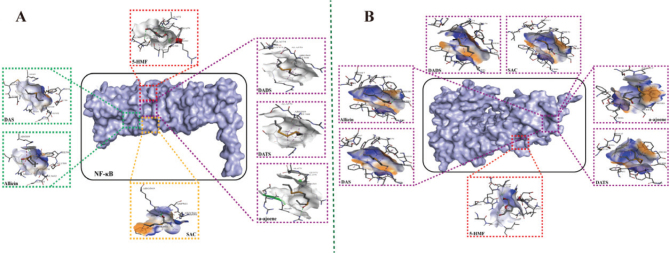
Direct interaction between aged garlic and NF-κB pathway. (A) Molecular docking analysis identifies potential binding regions between metabolites and NF-κB2. (B) Molecular docking analysis identifies potential binding regions between metabolites and NF-κB3. NF-κB: nuclear factor kappa-B.

**Fig. 4:**
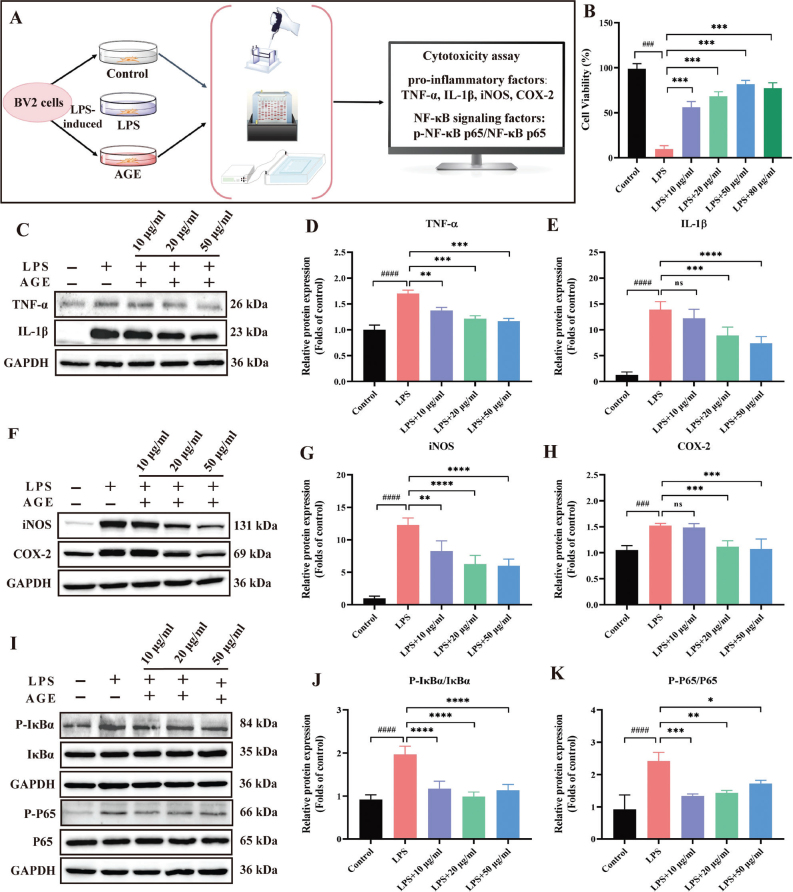
The effect of AGE on LPS-induced inflammation in vitro. (A) The procedure of the in vitro experimental. (B) Cell viability of AGE in LPS-induced BV-2 cells. (C–E) AGE inhibition of pro-inflammatory factors expression in LPS-stimulated BV-2 cells. (F–H) AGE inhibition of iNOS and COX-2 expression in LPS-stimulated BV-2 cells. (I–K) AGE Down-Regulated NF-κB Signaling Pathway in LPS-stimulated BV2 Cells. Data are mean ± SD (*n* = 3). ^#^*p* < 0.05, ^##^*p* < 0.01, ^###^*p* < 0.001, ^####^*p* < 0.0001 compared to control group. *p < 0.05, **p < 0.01, ***p < 0.001, ****p < 0.0001 compared to LPS group. LPS: Lipopolysaccharide; AGE: aged garlic extract; TNF-α, tumor necrosis factor-α; IL-1β: interleukin-1β; iNOS: inducible nitric oxide synthase; COX-2: cyclooxygenase-2.

### Construction and analysis of the AG-target-neuroinflammation network

Subsequently, the compound-target-pathway (CTP) network was established and depicted in Fig. 2F. Upon scrutinizing the network, it was discerned that the NF-κB signaling pathway exhibited high level of enrichment, thereby signifying its paramount significance as the most pertinent pathway.

### Molecular docking verification

The identification of significant targets is conducted by screening the PPI interaction network, followed by the validation of the corresponding chemical constituents through molecular docking. The targets NF-κB2 and NF-κB3 were deleted. During the aging process, allicin contributes to the characteristic flavor and taste of garlic. Allicin and other thiosulfinates are immediately decomposed to other compounds such as diallyl sulfide, diallyl disulfide, and diallyl trisulfide, dithiins, and ajoene (21). At the same time, γ-glutamylcysteines are converted to (R)-3-(Allylthio)-2-aminopropanoic acid (SAC) through its catabolism pathway other than the alliineallicin pathway. SAC was considered to be a major active ingredient in AG. The seven active components examined in this study demonstrated a favorable affinity towards the two targets, as evidenced by the data presented in Table S4. This finding provides additional evidence supporting the notion that the active components of AG, namely allicin, SAC, diallyl sulfide (DAS), diallyl disulfide (DADS), and diallyl trisulfide (DATS), possess a strong affinity towards crucial targets, thereby indicating their potential in exerting anti-inflammatory effects (Fig. 3). Based on the binding energies from the docking analysis, we found that several metabolites bind more stably with NF-κB3 compared to NF-κB2. Therefore, we preliminarily speculate that the metabolites in AG may exert effects on neuroinflammation through the NF-κB3 (p65) pathway.

### AGE reduces the neuroinflammatory-associated factors in LPS-stimulated BV2 cells

A 1:1 mixture of ethyl acetate extract (Fig. S1) and water extract (Fig. S2) was used for the cell experiments. The levels of pro-inflammatory factors were assessed using western blot analysis (Fig. 4A). AGE extract has no effect on cell activity at a certain concentration (Fig. 4B). Treatment with AGE at concentrations of 10 μg/mL, 20 μg/mL, and 50 μg/mL resulted in significant reductions in the levels of TNF-α, IL-1β (Figs. 4C–4E). Preliminary showed that different concentrations of AGE can inhibit expression of pro-inflammation in LPS-induced BV2 cells. The iNOS and cyclooxygenase-2 (COX-2) expression were increased by LPS addition, compared with that of the control, AGE reduced the LPS-induced iNOS and COX-2 expression with the increased indirubin concentration (Figs. 4F–4H).

### AGE down-regulated the NF-κB signaling in LPS-stimulated BV2 cells

To explore whether the NF-κB pathway mediated indirubin-mediated inhibition of the inflammatory response, the NF-κB and IκBα (recombinant inhibitory subunit of nuclear factor kappa-B Alpha) protein levels were determined by western blotting. The extents of P65 and IκBα phosphorylation were significantly increased after LPS treatment but were reduced by AGE pretreatment, in a dose-dependent manner (Figs. 4I–4K).

### Transcriptomic changes between the LPS-treated and control groups

Given that AGE influenced LPS-induced proinflammatory responses in vitro, we extended our investigation to assess the effects of AGE pretreatment on LPS-induced microgliosis in vivo. Based on metabolomic data, we found that AGE contains abundant metabolites in both low-polarity and high-polarity regions. Literature reports suggest that the hexane, chloroform and n-butanol of AG, a low-polarity solvent, has a protective effect in TNFα-induced Monocytic Cell through the NF-κB pathway (22). Meanwhile, the water extract of AG is rich in polysaccharides, which have shown more potent antioxidant and antibacterial effects (20).The water extract of AG is rich in polysaccharides, which have an immunomodulatory effect on LPS-induced RAW 264.7 macrophages (23).

**Fig. 5:**
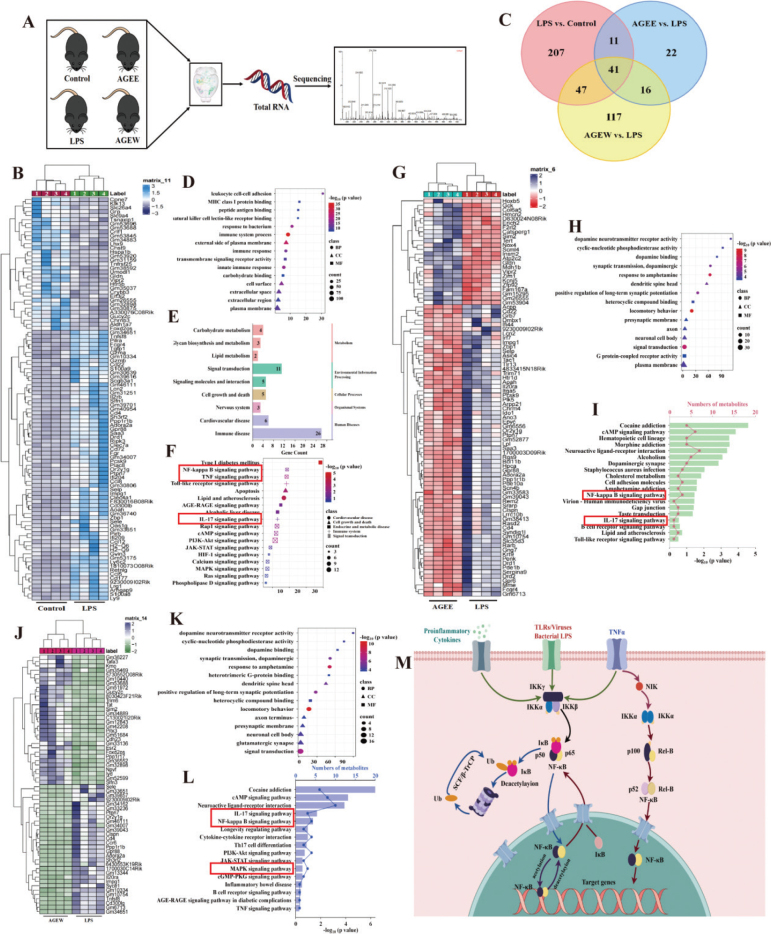
Transcriptomic differences in the brain between AGE and LPS-induced mice group. (A) The procedure of the in vitro experimental. (B) Venn diagram of the differential genes in these groups. (C) Heatmaps of the differential genes in LPS vs. control groups. (D) GO-enrichment analysis of differential genes. (E) The classification of the pathways. (F) KEGG enrichment of the 18 pathways related to inflammation. (G) Heatmaps of the differential genes in AGEE versus LPS groups. (H) GO-enrichment analysis of differential genes. (I) KEGG enrichment of the 16 pathways related to inflammation. (J) Heatmaps of the differential genes in AGEW vs. LPS groups. (K) GO-enrichment analysis of differential genes. (L) KEGG enrichment of the 16 pathways related to inflammation. (M) Proposed mechanistic of AGE against neuroinflammation. AGE: aged garlic extract; AGEE: aged garlic ethyl acetate extract; AGE W: aged garlic water extract; GO: Gene Ontology; KEGG: Kyoto Encyclopedia of Genes and Genomes.

To systematically understand the transcriptomic changes in mice, we performed transcriptome sequencing on the brains of mice treated with or without LPS and AGE (Fig. 5A). PCA revealed a clear distinction between the Con, LPS, and AGE groups (Fig. S3A). Differentially expressed genes (DEGs) were identified with a significance threshold of *p* < 0.05 and |log_2_FC|>1. In the comparison between LPS and control groups, 306 DEGs were identified, with 273 up-regulated and 33 down-regulated (Fig. S3B). Some of these DEGs are illustrated in Fig. 5B. Intersection analysis revealed 41 overlapping DEGs among the LPS vs. Control, AGE (E) vs. LPS, and AGE (W) vs. LPS comparisons (Fig. 5C). Pathway enrichment analysis of these DEGs highlighted major pathways involved in disease processes and drug treatment. GO enrichment analysis showed that, compared to the control group, LPS induction predominantly affected pathways related to immune system processes, innate immune responses, immune responses, and transmembrane signaling receptor activity (Fig. 5D). KEGG pathway enrichment analysis, shown in Fig. 5E, identified nine disease-related pathways, with immune-related pathways prominently featured (Fig. 5F). These include the NF-κB pathway, mitogen-activated protein kinase (MAPK) signaling pathway, and interleukin-17 (IL-17) pathway, which are consistent with our network pharmacology findings.

### AGE regulated the expression of inflammation-related genes

After treatment with AGE (E), a total of 90 differentially expressed genes (DEGs) were identified between the AGE (E) and LPS groups, with 24 up-regulated and 66 down-regulated (Fig. S3C). Some of these DEGs are shown in Fig. 5G. GO enrichment analysis of these DEGs revealed that, compared to the LPS-treated group, DEGs in the AGE (E) group were primarily associated with synaptic transmission, dopaminergic processes, response to amphetamine, signal transduction, and presynaptic membrane functions (Fig. 5H). KEGG pathway enrichment analysis (Fig. 5I) indicated significant enrichment of the NF-κB pathway, IL-17 pathway, and Toll-like receptor signaling pathway, suggesting that AGE (E) modulates LPS-induced inflammation in mice through the NF-κB pathway.

For the AGE (W) treatment, similar to AGE (E), a total of 221 DEGs were identified between AGE (W) and LPS groups, with 73 up-regulated and 148 down-regulated (Fig. S3D), and some of these DEGs are depicted in Fig. 5J. GO enrichment analysis for AGE (W) also showed enrichment in synaptic transmission, dopaminergic processes, response to amphetamine, signal transduction, and presynaptic membrane functions (Fig. 5K), which closely resembles the findings for AGE (E). KEGG pathway enrichment analysis for AGE (W) (Fig. 5L) highlighted the NF-κB pathway, IL-17 pathway, and MAPK signaling pathway, demonstrating that AGE (W) can similarly regulate LPS-induced inflammation in mice through the NF-κB pathway.

Pro-inflammatory cytokines such as IL-1β, IL-6, interleukin 10 (IL-10), and TNF-α are crucial mediators of both acute and chronic inflammatory responses (24). The primary regulatory transcription factor NF-κB forms homo- or hetero-dimers with P50 and P65 proteins, which are bound to the inhibitor IkBα. The dissociation of these complexes is triggered by factors such as cytokines, free radicals, stress, ultraviolet light, oxidized low-density lipoproteins, and bacterial or viral antigens. Upon activation, IkBα kinase phosphorylates NF-κB P65, leading to its ubiquitin-mediated degradation through the proteasome pathway (25). Once activated, NF-κB translocates to the nucleus, promoting the expression of various genes that regulate innate and adaptive immune responses, cell adhesion, inflammation, and anti-apoptotic processes (Fig. 5M).

### AGE relieved sickness and depressive-like behaviors in LPS-stimulated mice

**Fig. 6:**
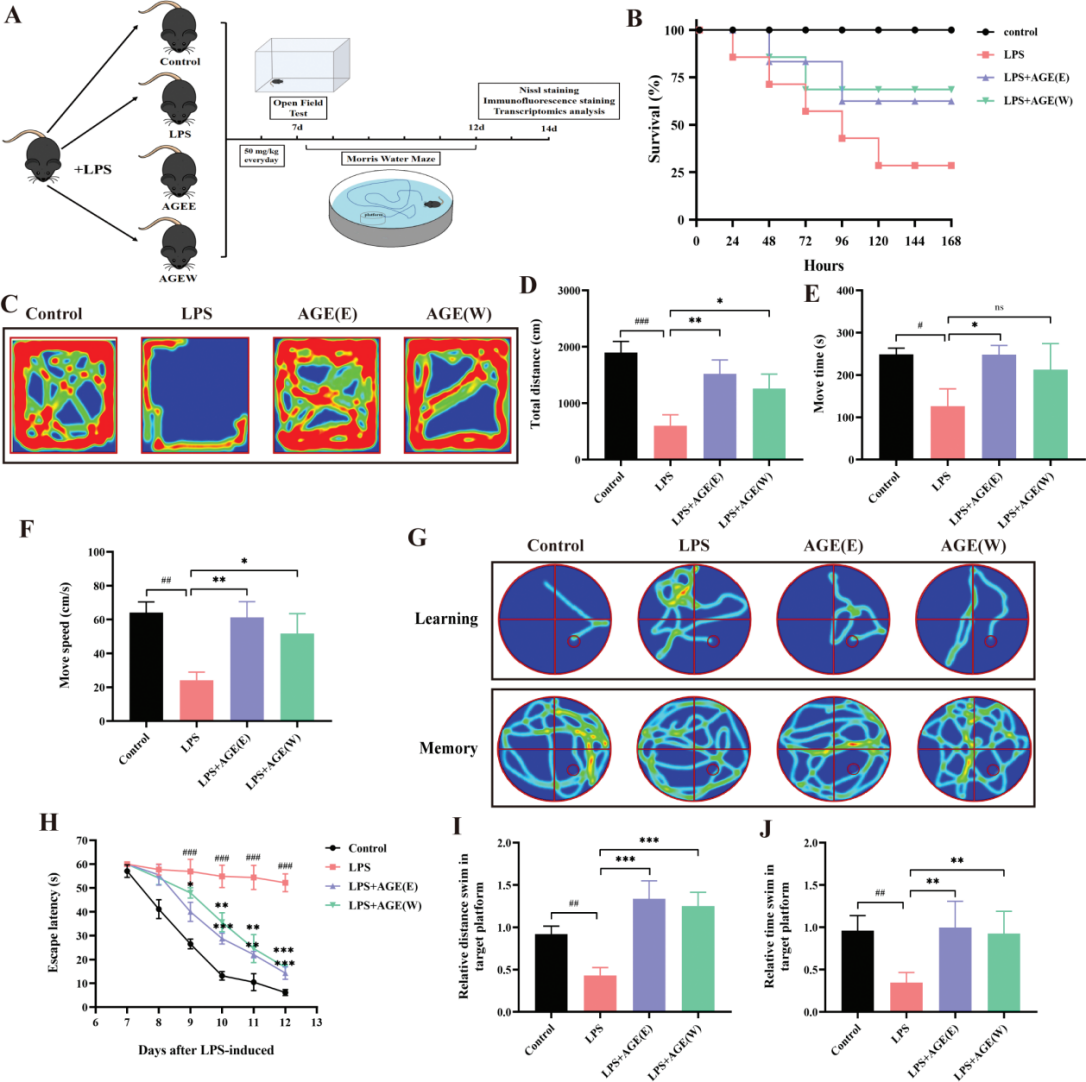
AGE improved sensorimotor and cognitive function, relieved sickness and depressive-like behaviors in LPS-stimulated mice. (A) The procedure of the in vitro experimental. (B) The effects of AGE on the LPS-induced mice using the Kaplan-Meier method to measure survival rates, *n* = 25. (C–F) The sickness and depressive-like behaviors in LPS-stimulated mice were measured by open field test, *n* = 6. (G) Representative traces of the mice from the maze latency trials (learning) and the swimming traces from trials (memory), *n* = 6. (H) The escape latency from day 7 to 12, *n* = 6. (I) The relative distance swim in target platform and (J) the relative time spent in the target quadrant, *n* = 6. Data are expressed as the mean ± SD. ^#^*p* < 0.05, ^##^*p* < 0.01, ^###^*p* < 0.001 compared to the control group. *p < 0.05, **p < 0.01, ***p < 0.001 compared to the LPS group. AGE: aged garlic extract; AGEE: aged garlic ethyl acetate extract; AGE W: aged garlic water extract; LPS: Lipopolysaccharide.

Subsequently, a battery of behavioral assessments was conducted to evaluate the impact of AG ethyl acetate extract (AGE (E)) and AG water extract (AGE (W)) on the restoration of sensorimotor and cognitive functions in LPS-stimulated mice (Fig. 6A). After LPS induction, the mortality rate of mice increased, while AGE treatment reduced the mortality rate of the mice (Fig. 6B). LPS has been established as a causative agent for the manifestation of pathological and depression-like behavior in mice. Therefore, we explored the effect of AGE on behavioral disorders through open-field experiments. Notably, the administration of AGE exhibited a significant ameliorating effect on LPS-induced motor activity reduction, as evidenced by a substantial increase in total distance covered and movement time, when compared to the LPS group (Figs. 6C–6F).

### AGE improved sensorimotor and cognitive function in LPS-stimulated mice

The water maze experiment revealed a notable rise in escape latency among LPS-stimulated mice in their pursuit of platforms, in contrast to the control group. However, treatment with AGE (E) and AGE (W) exhibited the ability to reverse this phenomenon, as evidenced by a decrease in escape latency from day 7 to day 12 following LPS stimulation, when compared to the LPS group (Figs. 6G–6J).

### AGE reduced neuronal injury and microglial activation in LPS-stimulated mice

**Fig. 7:**
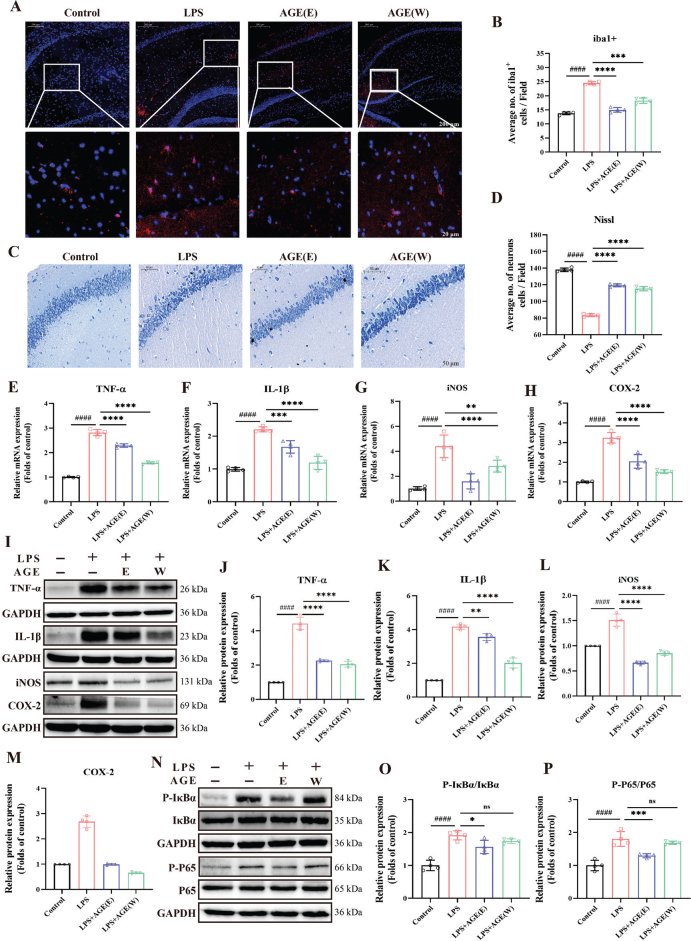
The effect of AGE on LPS-induced inflammation in vivo. (A) The area of iba1-positive cells. (B) Representative images of iba1-labeled activated microglia in the hippocampal. (C) Nissl staining of the brain in neuroinflammatory mice induced by LPS. (D) Average number of neurons cells. (E–H) The mRNA expression of TNF-α, IL-1β, iNOS and COX-2, *n* = 4. (I–M) The protein levels of TNF-α, IL-1β, iNOS and COX-2, *n* = 4. (N–P) AGE Down-Regulated NF-κB Signaling Pathway in LPS-stimulated mice, *n* = 4. Data are expressed as the mean ± SD. ^#^*p* < 0.05, ^##^*p* < 0.01, ^###^*p* < 0.001 compared to control group. *p < 0.05, **p < 0.01, ***p < 0.001 compared to LPS group. AGE: aged garlic extract; AGEE: aged garlic ethyl acetate extract; AGE W: aged garlic water extract; LPS: Lipopolysaccharide; TNF-α: tumor necrosis factor-α; IL-1β: interleukin-1β; iNOS: inducible nitric oxide synthase; COX-2: cyclooxygenase-2.

To examine the regulatory role of AGE on neuroinflammation, we examined the expression of Iba1^+^, which is used as a specific marker for microglia. Immunofluorescence staining showed an increase in Iba1-labeled microglia in the hippocampus of the LPS group compared with the control group. However, after AGE treatment, the trend of increase decreased (Figs. 7A–7B).

Nissl staining was used to identify apoptotic neurons in LPS-stimulated hippocampal. Under the light microscope, it was observed that the hippocampal neurons of control mice were arranged in an orderly manner, with normal cell morphology and structure, and Nissl bodies were large and obvious. The organization of neurons in the LPS-stimulated group appears to be disrupted, characterized by a scarcity of cells, diminished or indistinct Nissl bodies, and the presence of some cells exhibiting nuclear pyknosis. In contrast, the AGE (E) and AGE (W) groups exhibit a slight reduction in Nissl bodies, but overall maintain a morphology and structure that closely resemble the control group, resulting in reduced damage compared to the LPS-stimulated group. Notably, the AGE (W) group demonstrates a superior degree of damage mitigation when compared to the AGE (E) group, as depicted in Figs. 7C–7D.

### Effects of AGE on neuroinflammatory-associated factors and NF-κB pathway in LPS-stimulated mice

The levels of TNF-α, IL-1β, iNOS, and COX-2 in the brain of mice were assessed (Figs. 7E–7H). In the group induced with LPS, there was an observed increase in the levels of TNF-α, IL-1β, iNOS, and COX-2 specifically within the hippocampus. Treatment with AG ethyl acetate extract and water extract at a dosage of 50 mg/kg resulted in a significant reduction of these levels within the hippocampal tissues of mice. The Western blot results also showed that after LPS-induced neuroinflammation in mice, the protein expression of inflammatory factors such as TNF-α IL-1β, iNOS, and COX-2 in brain tissue was significantly increased. AGE significantly reduced the expression of related inflammatory factors (Figs. 7I–7M).

The treatment with AGE resulted in a significant decrease in the ratio of p-NF-κB p65/NF-κB p65. Furthermore, AGE also suppressed the phosphorylation levels of IκB, which are crucial upstream signals in the NF-κB signaling pathway (Figs. 7N–7P).

## Discussion

In this study, we demonstrated that AG exhibits potent neuroprotective effects by modulating the NF-κB signaling pathway. Our findings demonstrate that AG effectively reduces neuroinflammation, enhances neuronal survival, and improves cognitive function in LPS-induced neuroinflammatory models, suggesting its potential as a therapeutic agent for neuroinflammatory conditions.

The administration of AGE treatment resulted in enhanced sensorimotor and cognitive learning abilities, as evidenced by reduced escape latencies and path lengths. Subsequent investigations demonstrated that AGE exhibited potential anti-neuroinflammatory effects in BV-2 cells and C57BL/6 mice stimulated with LPS. Additionally, both AGE (E) and AGE (W) treatments mitigated hippocampal neuron damage and alleviated sickness and depressive-like behaviors in LPS-stimulated mice, as assessed through the open field test. The neuroprotective effects of advanced glycation end products may be attributed to their ability to inhibit the activation of the NF-κB signaling pathway, leading to a reduction in the secretion of TNF-α and IL-1β in both LPS-induced BV2 cells and mice.

Garlic is widely used as a condiment, and it has many health benefits (26). However, the pungent smell makes it difficult for people to accept it. Garlic is a kind of food with high potential antioxidant activity, which is enhanced by processing. Among them, the antioxidant activity of AG was meaningfully higher than garlic, which was due to the higher polyphenol content and scavenging activity of AG (8). AG is a variety of garlic that is devoid of any discernible odor, and is obtained through the process of fermenting raw garlic (27). The content of compounds in AG is related to the heat treatment conditions. Studies have reported that many anti-disease effective ingredients in AG increased, especially polyphenols, flavonoids, and some intermediates of the Millard reaction are considered as agents possessing antioxidant properties (28, 29). Meanwhile, the research reported that AGE can inhibit NF-κB transcription factor in TNF-α-stimulated HUVECs (22). Despite the numerous benefits of AG for human health, there is limited information available regarding its neuroprotective activity.

Neuroinflammation, a significant pathological process observed in numerous neurodegenerative diseases (30), is influenced by systemic inflammation and evidenced by elevated levels of pro-inflammatory cytokines as well as local neuronal damage. Extensive research has been conducted on neuroinflammation in recent years, establishing it as a characteristic feature of central nervous system (CNS) disorders, including ischemic stroke and temporal lobe epilepsy (31).

Physiologically, the brain responds with neuroinflammation as a protective response, but when this reaction is overdone, it is harmful. In reality, it inhibits the regeneration of neurons (32). Under normal circumstances, microglia protect the brain from pathogens and help maintain tissue homeostasis. Pathological damage, whether induced by endogenous or exogenous stimulation, can prompt microglia to transition into an ‘activated’ state, altering their morphology to facilitate phagocytic activity and releasing a range of pro-inflammatory or cytotoxic factors, including TNF-α, IL-1β, IL-6, among others (33). The accumulation of these pro-inflammatory factors subsequently results in the impairment and deterioration of neighboring neurons. In this study, we verified that AGE inhibits the occurrence of neuroinflammation by suppressing pro-inflammatory factors, including TNF-α and IL-1β, thereby improving learning and cognitive function in mice and protecting brain tissue.

Network pharmacology is an interdisciplinary field that integrates principles from pharmacology, bioinformatics, and network science to understand the complex interactions between biological systems and drug compounds (34). Unlike traditional pharmacology, which often focuses on individual drug–target interactions, network pharmacology takes a holistic approach by considering the entire biological network. This approach recognizes the fact that diseases are not caused by the dysfunction of a single gene or protein but rather by the dysregulation of interconnected molecular pathways. In network pharmacology studies, a network-based framework is employed to model and analyze the relationships between drugs, targets, diseases, and biological pathways. The strength of network pharmacology lies in its ability to unveil the multifaceted mechanisms of the action of drugs and their impact on biological systems. By examining the connectivity patterns within these networks, researchers can identify key nodes or hub molecules that play crucial roles in mediating the effects of drugs on specific diseases. This holistic perspective allows for a more comprehensive understanding of the polypharmacological nature of drugs, enabling the exploration of potential therapeutic targets and the discovery of novel treatment strategies (35).

Multiple studies have demonstrated the involvement of the NF-κB pathway in the pathogenesis of neuroinflammation, whereby its activation can elicit an inflammatory response (36). In recent years, activation of the NF-κB pathway has been recognized as a strategy for alleviating neuroinflammatory diseases. NF-κB is a pathway closely related to neuroinflammatory responses. There is increasing evidence that many neurological diseases are related to the inflammatory response, such as ischemic stroke, intracranial hemorrhagic disease, depression (37). The network pharmacology results show that AGE inhibits the occurrence of neuroinflammation through the NF-κB pathway, providing reliable theoretical guidance for our study.

Various compounds in AG may exhibit varying polarities. To achieve a more comprehensive composition, we employed ethyl acetate extract and water as extraction solvents to isolate the lipophilic and water-soluble constituents of AG. AGE (E) denotes the ethyl acetate extract of AG, primarily comprising compounds possessing lower polarity, such as garlic oil. AGE (W) is the water extract of AG, which is mainly composed of compounds with higher polarity. We then induce neuroinflammation with LPS because it activates glial cells to synthesize and secrete pro-inflammatory cytokines-interleukin (IL-1), TNF-α, involved in the pathological process of neuroinflammation (38). IL-1β and TNF-α were detected after LPS administration as markers of neuroinflammation. The findings of this study indicate that the administration of AGE treatment resulted in a decrease in pro-inflammatory factors and mediators in BV2 cells and mice that were stimulated with LPS. Additionally, it was observed that AGE treatment led to a significant reduction in the presence of NF-κB P65.

## Conclusions

In the present study, we utilized integrated multi-omics, network pharmacology, and experimental validation to investigate the therapeutic effects and underlying mechanisms of AGE in neuroinflammation. Our findings suggest that AGE may have a neuroprotective effect against LPS-induced neuroinflammation. AGE effectively reduced the levels of inflammatory factors and mitigated the activation of the NF-κB signaling pathway. Overall, these results indicate that AGE holds promise as a potential anti-neuroinflammatory agent.

## Supplementary Material


