# Supplementary material for: Aged garlic attenuates neuroinflammation via modulating the NF-κB pathway: Insights from multi-omics analyses
Source: Food Nutr Res. 2025 Jul 9;69:10.29219/fnr.v69.11923. doi: 10.29219/fnr.v69.11923 (PMC12320767; doi:10.29219/fnr.v69.11923)
Supplement: Supplementary file 1 [file FNR-69-11923-s1.doc]

**Legends to supplementary material**

**Table S1.** Compositions of experimental diets (g/kg).

**Table S2.** Primer Sequences for RT-PCR.

**Table S3.** Pharmacological and molecular properties data.

**Table S4.** Molecular docking result of binding energies of seven active compounds with NF-κB2 and NF-κB3 target genes.

**Figure S1.** HPLC chromatogram of ethyl acetate extract.

**Figure S2.** HPLC chromatogram of water extract.

**Figure S3.** Transcriptomic differences in the brain between AGE and LPS-induced mice group. (A) PCA plot among the three groups. (B) Volcano plots of differential metabolites in LPS and control groups. (D) Volcano plots of differential metabolites in AGE(E) and LPS groups. (E) Volcano plots of differential metabolites in AGE(W) and LPS groups.

| **Table S1**. Compositions of experimental diets (g/kg). | |
| --- | --- |
| **Ingredient** | **Content (g/kg)** |
| Cornstarch | 390 |
| Casein | 200 |
| Sucrose | 100 |
| Dextrin | 138 |
| Soybean oil | 70 |
| Cellulose | 50 |
| Mineral mix | 35 |
| Vitamin mix | 10 |
| L-Cystine | 3 |
| Choline bitartrate | 3 |
| Carbohydrates | 390 |
| Protein | 210 |
| Brute fiber | 50 |
| Total | 1000 |
| Mineral and vitamin are based on the AIN-93M vitamin and mineral mixes. | |

| **Table S2.** Primer Sequences for RT-PCR. | | |
| --- | --- | --- |
| **Primer** | **Forward** | **Reverse** |
| TNF-α | GGACTAGCCAGGAGGGAGAACAG | GCCAGTGAGTGAAAGGGACAGAAC |
| IL-1β | TCGCAGCAGCACATCAACAAGAG | AGGTCCACGGGAAAGACACAGG |
| iNOS | GTGTCAGTGGCTTCCAGCTC | CTCATGCGGCCTCCTTTGAG |
| COX-2 | GTGCCTGGTCTGATGATGTATGC | GGATGCTCCTGCTTGAGTATGTC |

| **Table S3.** Pharmacological and molecular properties data. | | | | | | | | | | | |
| --- | --- | --- | --- | --- | --- | --- | --- | --- | --- | --- | --- |
| [**Mol ID**](https://old.tcmsp-e.com/tcmspsearch.php?qr=Allii Sativi Bulbus&qsr=herb_en_name&token=c7586c39ef85e02a2cc75e07d2e10bbf) | [**Molecule Name**](https://old.tcmsp-e.com/tcmspsearch.php?qr=Allii Sativi Bulbus&qsr=herb_en_name&token=c7586c39ef85e02a2cc75e07d2e10bbf) | [**MW**](https://old.tcmsp-e.com/tcmspsearch.php?qr=Allii Sativi Bulbus&qsr=herb_en_name&token=c7586c39ef85e02a2cc75e07d2e10bbf) | [**AlogP**](https://old.tcmsp-e.com/tcmspsearch.php?qr=Allii Sativi Bulbus&qsr=herb_en_name&token=c7586c39ef85e02a2cc75e07d2e10bbf) | [**Hdon**](https://old.tcmsp-e.com/tcmspsearch.php?qr=Allii Sativi Bulbus&qsr=herb_en_name&token=c7586c39ef85e02a2cc75e07d2e10bbf) | [**Hacc**](https://old.tcmsp-e.com/tcmspsearch.php?qr=Allii Sativi Bulbus&qsr=herb_en_name&token=c7586c39ef85e02a2cc75e07d2e10bbf) | [**OB (%)**](https://old.tcmsp-e.com/tcmspsearch.php?qr=Allii Sativi Bulbus&qsr=herb_en_name&token=c7586c39ef85e02a2cc75e07d2e10bbf) | [**Caco-2**](https://old.tcmsp-e.com/tcmspsearch.php?qr=Allii Sativi Bulbus&qsr=herb_en_name&token=c7586c39ef85e02a2cc75e07d2e10bbf) | [**BBB**](https://old.tcmsp-e.com/tcmspsearch.php?qr=Allii Sativi Bulbus&qsr=herb_en_name&token=c7586c39ef85e02a2cc75e07d2e10bbf) | [**DL**](https://old.tcmsp-e.com/tcmspsearch.php?qr=Allii Sativi Bulbus&qsr=herb_en_name&token=c7586c39ef85e02a2cc75e07d2e10bbf) | [**FASA-**](https://old.tcmsp-e.com/tcmspsearch.php?qr=Allii Sativi Bulbus&qsr=herb_en_name&token=c7586c39ef85e02a2cc75e07d2e10bbf) | [**HL**](https://old.tcmsp-e.com/tcmspsearch.php?qr=Allii Sativi Bulbus&qsr=herb_en_name&token=c7586c39ef85e02a2cc75e07d2e10bbf) |
| MOL008359 | [DATS/diallyl trisulfide](https://old.tcmsp-e.com/molecule.php?qn=8359) | 210.44 | 3.8 | 0 | 0 | 49.42 | 1.82 | 2.03 | 0.01 | 0.55 | 8.2 |
| MOL004046 | [Dimethyl tetrasulfide](https://old.tcmsp-e.com/molecule.php?qn=4046) | 158.36 | 2.57 | 0 | 0 | 51.34 | 2.02 | 2.14 | 0 | 0 | 12.18 |
| MOL008365 | [S-Methylmethionine](https://old.tcmsp-e.com/molecule.php?qn=8365) | 164.37 | 3.59 | 0 | 0 | 62.95 | 1.79 | 1.91 | 0.01 | 0.3 | 5.48 |
| MOL008363 | [Methylallyl trisulfide](https://old.tcmsp-e.com/molecule.php?qn=8363) | 152.33 | 2.59 | 0 | 0 | 23.2 | 1.92 | 2.19 | 0 | 0.54 |  |
| MOL008361 | [3-METHYL-2-THIABUTANE](https://old.tcmsp-e.com/molecule.php?qn=8361) | 90.21 | 1.52 | 0 | 0 | 31.49 | 1.67 | 2.18 | 0 | 0.36 | 11.79 |
| MOL007619 | [Methyl allyl sulfide](https://old.tcmsp-e.com/molecule.php?qn=7619) | 88.19 | 1.41 | 0 | 0 | 70.09 | 1.6 | 1.84 | 0 | 0.44 | 12.01 |
| MOL008358 | [Oil garlic](https://old.tcmsp-e.com/molecule.php?qn=8358) | 114.23 | 2.03 | 0 | 0 | 74.81 | 1.65 | 1.91 | 0.01 | 0.4 | 11.61 |
| MOL008354 | [allicin](https://old.tcmsp-e.com/molecule.php?qn=8354) | 162.3 | 1.51 | 0 | 1 | 78.41 | 0.98 | 1.22 | 0.01 | 0.43 | 4.83 |
| MOL007601 | [(+)-L-Alliin](https://old.tcmsp-e.com/molecule.php?qn=7601) | 177.25 | -0.83 | 3 | 4 | 86.68 | -0.57 | -1.04 | 0.02 | 0.38 | 5.07 |
| MOL007630 | Propyl Trisulfide | 182.41 | 3.72 | 0 | 0 | 40.68 | 1.81 | 1.91 | 0.01 | 0.37 | 5.54 |
| MOL007628 | [Methyl trisulfide](https://old.tcmsp-e.com/molecule.php?qn=7628) | 126.29 | 1.98 | 0 | 0 | 10.72 | 2.01 | 2.27 | 0 | 0.58 |  |
| MOL006791 | Epigallocatechin | 306.29 | 1.65 | 6 | 7 | 24.18 | -0.22 | -0.82 | 0.27 | 0.33 |  |
| MOL002516 | Zingerone | 194.25 | 1.63 | 1 | 3 | 25.23 | 0.87 | 0.48 | 0.05 | 0.29 |  |
| MOL000771 | P-Coumaric Acid | 164.17 | 1.64 | 2 | 3 | 43.29 | 0.46 | 0.13 | 0.04 | 0.45 | 4.43 |
| MOL012744 | Resveratrol | 228.26 | 3.01 | 3 | 3 | 19.07 | 0.8 | -0.01 | 0.11 | 0.49 |  |
| MOL011865 | Rosmarinic Acid | 360.34 | 2.69 | 5 | 8 | 1.38 | -0.54 | -1.24 | 0.35 | 0.47 |  |
| MOL000492 | epicatechin | 290.29 | 1.92 | 5 | 6 | 54.83 | -0.03 | -0.73 | 0.24 | 0 | 0.61 |
| MOL001002 | Ellagic Acid | 302.2 | 1.48 | 4 | 8 | 43.06 | -0.44 | -1.41 | 0.43 | 0.43 | -1.04 |
| MOL000874 | Paeonol | 166.19 | 1.29 | 1 | 3 | 28.79 | 0.93 | 0.84 | 0.04 | 0.32 |  |
| MOL000105 | Protocatechuic Acid | 154.13 | 0.9 | 3 | 4 | 25.37 | 0.1 | -0.17 | 0.04 | 0.43 |  |
| MOL000098 | Quercetin | 302.25 | 1.5 | 5 | 7 | 46.43 | 0.05 | -0.77 | 0.28 | 0.38 | 14.4 |
| MOL000360 | Ferulic Acid | 194.2 | 1.62 | 2 | 4 | 39.56 | 0.47 | -0.03 | 0.06 | 0.34 | 2.38 |
| MOL010545 | 2-methoxycinnamic acid | 178.2 | 1.89 | 1 | 3 | 31.79 | 0.7 | 0.63 | 0.05 | 0.35 | 4.42 |
| MOL003178 | gentisinic acid | 154.13 | 0.9 | 3 | 4 | 29.33 | 0.26 | 0.02 | 0.04 | 0.45 | 4.12 |
| MOL007189 | mesaconic acid | 130.11 | 0.44 | 2 | 4 | 69.77 | -0.3 | -0.53 | 0.02 | 0.38 | 11.8 |
| MOL000384 | glucuronic acid | 194.16 | -2.47 | 5 | 7 | 3.35 | -1.91 | -4.72 | 0.04 | 0 |  |
| MOL010768 | kynurenic acid | 189.18 | 1.78 | 2 | 4 | 44.72 | 0.33 | -0.03 | 0.08 | 0.41 | 5.98 |
| MOL001477 | Tyramine | 137.2 | 0.99 | 3 | 2 | 45.11 | 0.74 | 0.52 | 0.02 | 0.32 | -2.52 |
| MOL000067 | Valine | 117.17 | 0.24 | 3 | 3 | 53.33 | 0.04 | -0.14 | 0.01 | 0 | 11.34 |
| MOL000068 | Isoleucine | 131.2 | 0.7 | 3 | 3 | 59.05 | 0.06 | -0.11 | 0.02 | 0 | 11.21 |
| MOL000429 | Asparagine | 132.14 | -1.85 | 5 | 5 | 83.96 | -0.88 | -1.15 | 0.02 | 0 | 11.59 |
| MOL000065 | Aspartic acid | 133.12 | -1.25 | 4 | 5 | 79.74 | -1.02 | -1.53 | 0.02 | 0 | 11.38 |
| MOL009676 | Glutamine | 146.17 | -1.53 | 5 | 5 | 87.9 | -0.96 | -1.47 | 0.02 | 0.35 | 11.34 |
| MOL000055 | Lysine | 146.22 | -0.68 | 5 | 4 | 29.33 | -0.66 | -1.44 | 0.02 | 0 |  |
| MOL000052 | Glutamic acid | 147.15 | -0.92 | 4 | 5 | 6.66 | -1.05 | -1.97 | 0.02 | 0 |  |
| MOL000041 | Phenylalanine | 165.21 | 0.96 | 3 | 3 | 41.62 | 0.36 | 0.22 | 0.04 | 0 | 4.62 |
| MOL001780 | Tryptophan | 204.25 | 1.25 | 4 | 3 | 75.93 | 0.26 | -0.17 | 0.08 | 0.27 | -2.49 |
| MOL000054 | Arginine | 174.24 | -1.11 | 7 | 6 | 47.64 | -0.49 | -1.04 | 0.03 | 0 | 0.85 |
| MOL000388 | Aminobutyric acid | 103.14 | -0.62 | 3 | 3 | 24.09 | -0.26 | -0.57 | 0.01 | 0 |  |
| MOL003795 | Pipecolic acid | 129.18 | 0.4 | 2 | 3 | 66.14 | 0.32 | 0.38 | 0.02 | 0.28 | 11.08 |
| MOL003800 | Citrulline | 175.22 | -1.32 | 6 | 6 | 52.96 | -0.86 | -1.46 | 0.03 | 0.31 | 0.9 |
| MOL001477 | Tyramine | 137.2 | 0.99 | 3 | 2 | 45.11 | 0.74 | 0.52 | 0.02 | 0.32 | -2.52 |
| MOL002321 | Oxoproline | 129.13 | -0.67 | 2 | 4 | 96.25 | -0.2 | -0.26 | 0.02 | 0.34 | 11.35 |
| MOL010246 | Flavonol | 238.25 | 2.57 | 1 | 3 | 47.91 | 1.02 | 0.77 | 0.16 | 0.45 | 19.49 |
| MOL007930 | Hesperidin | 610.62 | -0.48 | 8 | 15 | 13.33 | -2.03 | -2.7 | 0.67 | 0.31 |  |
| MOL000737 | Morin | 302.25 | 1.5 | 5 | 7 | 46.23 | 0 | -0.77 | 0.27 | 0.41 | 15.51 |
| MOL000481 | Genistein | 270.25 | 2.07 | 3 | 5 | 17.93 | 0.43 | -0.4 | 0.21 | 0 |  |
| MOL000422 | Kaempferol | 286.25 | 1.77 | 4 | 6 | 41.88 | 0.26 | -0.55 | 0.24 | 0 | 14.74 |
| MOL000006 | Luteolin | 286.25 | 2.07 | 4 | 6 | 36.16 | 0.19 | -0.84 | 0.25 | 0.39 | 15.94 |
| MOL004054 | isokobusone | 222.36 | 2.3 | 1 | 2 | 39.63 | 0.67 | 0.51 | 0.11 | 0 |  |
| MOL000416 | lariciresinol | 360.44 | 2.46 | 3 | 6 | 5.53 | 0.27 | -0.49 | 0.38 | 0 |  |
|  | Desoxycorticosterone | 331.2297788 | 2.86 | 0 | 0 | 0.23 | 0.31 | -3.57 | 0.26 | 0 | 4..81 |
| MOL006617 | kushenol f | 424.53 | 5.56 | 4 | 6 | 18.85 | 0.45 | -0.31 | 0.61 | 0.33 |  |
| MOL012951 | cyclic guanosine monophosphate | 345.24 | -1.98 | 5 | 11 | 3.76 | -1.39 | -1.96 | 0.4 | 0.39 |  |
| MOL011169 | ergosterol peroxide | 428.72 | 6.73 | 1 | 3 | 44.39 | 0.86 | 0.43 | 0.82 | 0.24 | 4.06 |
| MOL007716 | acetylshikonin | 330.36 | 2.77 | 2 | 6 | 62.39 | 0.3 | -0.31 | 0.27 | 0.35 | 29.08 |
| MOL002522 | (-)-citronellol | 156.3 | 3.05 | 1 | 1 | 38.05 | 1.19 | 1.12 | 0.02 | 0.24 | 5.19 |
| MOL006841 | harman | 182.24 | 2.46 | 1 | 1 | 33.1 | 1.52 | 1.53 | 0.1 | 0.29 | 9.15 |

| **Table S4.** Molecular docking result of binding energies of seven active compounds with NF-κB2 and NF-κB3 target genes. | | | | |
| --- | --- | --- | --- | --- |
| **Ingredient** | **Chemical formula** | **Molecular Weight** | **Target** | **Target Binding Energy/ (kcal·**mol-1) |
| 5-HMF | C6H6O3 | 126.11 | NF-κB2 | -4.5 |
|  |  |  | NF-κB3 | -4.7 |
| Allicin | C6H10OS2 | 162.27 | NF-κB2 | -3.1 |
|  |  |  | NF-κB3 | -4.3 |
| DADS | C6H10S2 | 146.27 | NF-κB2 | -3.1 |
|  |  |  | NF-κB3 | -3.7 |
| DAS | C6H10S | 114.21 | NF-κB2 | -3.1 |
|  |  |  | NF-κB3 | -3.7 |
| DATS | C6H10S3 | 178.33 | NF-κB2 | -3.6 |
|  |  |  | NF-κB3 | -2.9 |
| E-ajoene | C9H14OS3 | 234.39 | NF-κB2 | -3.2 |
|  |  |  | NF-κB3 | -4.3 |
| SAC | C6H11NO2S | 161.22 | NF-κB2 | -4.3 |
|  |  |  | NF-κB3 | -3.6 |
| 5-HMF, 5-hydroxymethyl furfural; DADS, diallyl disulfide; DAS, allyl sulfide; DATS, diallyl trisulfide; SAC, (R)-3-(Allylthio)-2-aminopropanoic acid. | | | | |

**Fig. S1.** HPLC chromatogram of ethyl acetate extract.

**Fig. S2.** HPLC chromatogram of water extract.


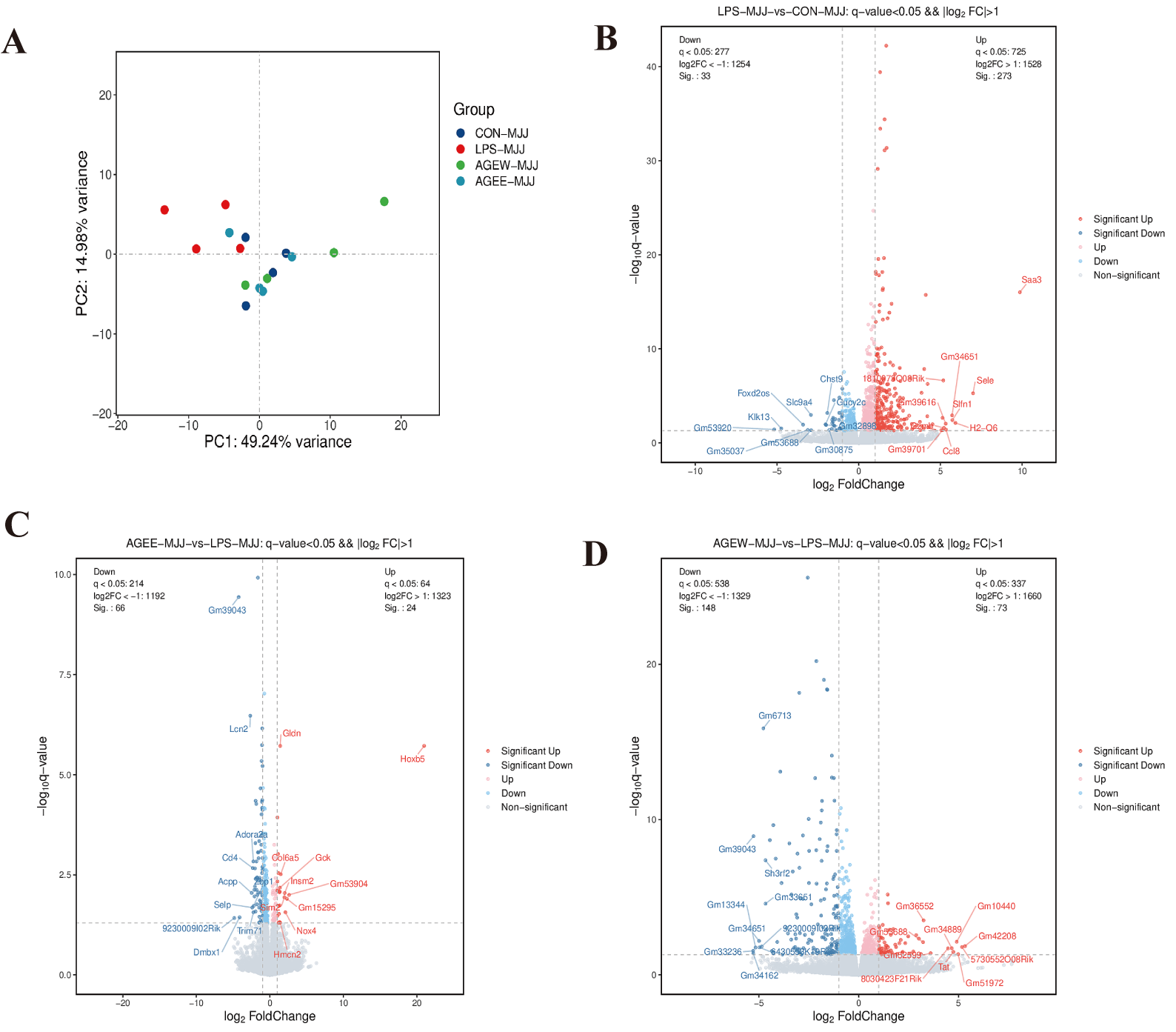


**Fig. S3.** Transcriptomic differences in the brain between AGE and LPS-induced mice group. (A) PCA plot among the three groups. (B) Volcano plots of differential metabolites in LPS and control groups. (D) Volcano plots of differential metabolites in AGE(E) and LPS groups. (E) Volcano plots of differential metabolites in AGE(W) and LPS groups.
